# Exploring Values Clarification and Health-Literate Design in Patient Decision Aids: A Qualitative Interview Study

**DOI:** 10.1177/0272989X251334356

**Published:** 2025-05-14

**Authors:** Julie Ayre, Hazel Jenkins, Richie Kumarage, Kirsten J. McCaffery, Christopher G. Maher, Mark J. Hancock

**Affiliations:** Sydney Health Literacy Lab, Sydney School of Public Health, Faculty of Medicine and Health, The University of Sydney, Sydney, NSW, Australia; Macquarie University Spinal Pain Research Centre, Department of Chiropractic, Faculty of Medicine, Health and Human Sciences, Macquarie University, Sydney, NSW, Australia; Sydney Health Literacy Lab, Sydney School of Public Health, Faculty of Medicine and Health, The University of Sydney, Sydney, NSW, Australia; Sydney Health Literacy Lab, Sydney School of Public Health, Faculty of Medicine and Health, The University of Sydney, Sydney, NSW, Australia; Institute for Musculoskeletal Health, Faculty of Medicine and Health, The University of Sydney and Sydney Local Health District, Sydney, NSW, Australia; Sydney School of Public Health, Faculty of Medicine and Health, The University of Sydney, Sydney, NSW, Australia; Macquarie University Spinal Pain Research Centre, Department of Health Professions, Faculty of Medicine, Health and Human Sciences, Macquarie University, Sydney, NSW, Australia

**Keywords:** health literacy, shared decision-making, patient decision aid, values clarification

## Abstract

**Background:**

This study explores patient and clinician perceptions of a patient decision aid, focusing on 2 features that are often absent: a health-literate approach (e.g., using plain language, encouraging question asking) and a tool that explicitly shows how treatment options align with patient values. The aim was to gather qualitative feedback from patients and clinicians to better understand how such features might be useful in guiding future decision aid development.

**Methods:**

We present a secondary analysis of data collected during the development of a decision aid for patients considering surgery for sciatica (20 patients with sciatica or low-back pain; 20 clinicians). Patient and clinician feedback on the design was collected via semi-structured interviews with a think-aloud protocol. Transcripts were analyzed using framework analysis.

**Results:**

Theme 1 explored designs that reinforced key messages about personal autonomy, including an interactive values-clarification tool. Theme 2 explored how participants valued encouragement and scaffolding to ask questions. Theme 3 described how patients preferred information they felt was complete, balanced, and understandable.

**Limitations:**

Further experimental and observational research is needed to quantitatively evaluate these decision aid features including evaluation among patients with and without low health literacy.

**Conclusions:**

A health-literate approach to decision aid design and embedding an interactive values-clarification tool may be useful strategies for increasing patient capacity to engage in key aspects of shared decision making. These features may support patients in developing an understanding of personal autonomy in the choice at hand and confidence to ask questions.

**Implications:**

Findings presented here were specific to the clinical context but provide generalizable practical insights for decision aid developers. This study provides insight into potential future areas of research for decision aid design.

**Highlights:**

- This qualitative study explored clinician and patient perceptions of health literacy features and an interactive values-clarification task within a decision aid for patients considering surgery for sciatica.
- The first theme described how patients and clinicians appreciated sections of the decision aid that reinforced the importance of personal choice. Patients and clinicians thought the interactive values-clarification task would help patients reflect on their values and support shared decision-making discussions.
- The second theme described how patients and clinicians appreciated strategies to encourage patients to ask questions of the surgeon.
- The third theme described patients’ preference for information that they felt was complete, balanced, and understandable.

## Introduction

Shared decision making describes a process of patients and clinicians working together to make a health decision that considers the medical evidence about a health issue, including the benefits and harms of different treatment options and the patient’s personal values and preferences.^1^ This process is particularly important when the evidence does not recommend one treatment over another and the treatment options vary in terms of their potential benefits and harms.^2^

Shared decision-making discussions can be enhanced by patient decision aids. These are tools that provide balanced, evidence-based information to help patients think about which benefits and harms matter most to them.^3^ A systematic review of 209 trials of patient decision aids for various health conditions showed that decision aids improve people’s knowledge of their options and help them feel better informed and clearer about what matters most to them.^4^ Values-clarification tasks are a common and recommended component of patient decision aids that aim to help patients come to a decision that aligns with their values (a “values-congruent decision”).^5^ Recent evidence has found that some of the most common values-clarification tasks such as asking people to consider pros and cons and rating the importance of different values do not achieve this goal.^6,7^ Instead, evidence suggests that tasks that give explicit feedback about how personal values align (or do not align) with a decision may be more effective.^6,7^ Surprisingly few studies have explored different ways that patient decision aid designs can deliver explicit feedback on the alignment between values and decisions or investigated patient attitudes toward these kinds of tasks.

Many patient decision aids have also failed to incorporate health literacy principles. A recent systematic review reported that only 12% of 213 patient decision aid trials addressed the needs of people with low health literacy or socially disadvantaged groups.^8^ This is an important oversight as people with low health literacy may be systematically excluded from opportunities for high-quality shared decision making if patient decision aids are too difficult to understand. Muscat et al.^9^ took this further, arguing that simplifying information is not enough and that shared decision-making resources must also help patients communicate effectively with clinicians. This can be achieved by fostering the skills and confidence to ask questions, reflect on personal values, and share these values with clinicians.

We sought to address these 2 gaps in patient decision aid research using the example of patients considering surgery for sciatica. Systematic reviews have concluded that while surgery is likely to speed up recovery of the sciatic pain, it will not improve a patient’s long-term outcomes compared with nonsurgical management such as medicines or physical therapy.^10–14^ They also report substantial uncertainty in the evidence base due to a limited number of high-quality studies. A few decision aids on this topic already exist. However, those that have incorporated the most recent trial evidence do not address the above gaps in health-literate design^15,16^ and values-clarification strategies.^15–17^

We developed a new decision aid for this patient group that was sensitive to health literacy needs and incorporated a novel values-clarification tool that gives explicit feedback on values alignment. The decision aid was co-designed with clinicians and people with lived experience and then iteratively refined through user testing.^18^ During user-testing interviews, many participants engaged in broader discussions about decision aids that went beyond how content specific to sciatica management was phrased and formatted. As such, in this article, we applied a broader lens to the data and aimed to explore clinician and patient perceptions of health-literate and values-clarification features that may be useful in guiding future decision aid development across varied health conditions.

## Methods

### Study Design

This study used semi-structured interviews with patients and clinicians to refine a prototype decision aid. This included a “think-aloud” protocol, with iterative revisions taking place across 7 rounds of feedback. Ethical approval for the study was obtained from the University of Sydney Human Research Ethics Committee (project No. 2022/678). The Consolidated Criteria for Reporting Qualitative Studies (COREQ) checklist is available in Appendix 1.

### Prototype Patient Decision Aid

Detail about the design process and iterative user testing are described in more detail elsewhere.^18^ The decision aid’s target patient group was people with sciatica caused by lumbar disc herniation with sciatic leg pain lasting less than 6 mo and with a referral to see a surgeon. Target clinicians were general practitioners (GPs), chiropractors, physiotherapists, and surgeons. The key decision under consideration was choosing between surgery or trying other options first (delaying the decision to have surgery to see if the pain resolved with time or nonsurgical management).

The design and content were informed by patient decision aid, health literacy, risk communication guidelines and literature,^5,7,8,19–25^ and systematic reviews on the topic.^10–14^ For example, the Patient Education Materials Assessment Tool^24^ is a checklist of health literacy principles that can be used when developing health resources. Muscat et al.^9^ also provided suggestions about how decision aids might foster core health literacy skills that help a person interact effectively with clinicians and integrate health information with their own personal values. A summary of health literacy principles incorporated into the decision aid is shown in Table 1. The initial design and content were further supported by a stakeholder working group (*N* = 16) comprising patients and clinicians.

**Table 1:** Examples of Health Literacy Principles^a^ Incorporated into the Decision Aid

Health Literacy Principle	Example in Decision Aid
**Clearly stated purpose of the resource**	Introduction gives an overview of the different sections (before, during, and after the consultation).
**Language:** Use common, everyday language and active voice. Limit jargon and define medical terms the first time they are used.	The grade reading score of the decision aid prototype was 9.5, or grade 8.1, with the words *surgery* and *sciatica* removed. This reading level was maintained in the final version (grade 9.0, or 7.7 with the 2 key terms removed). Medical jargon was used sparingly. The Health Literacy Editor^22^ estimated 11% of the text was complex (words with simpler alternatives, words uncommon in English, or acronyms). The lexical density was 3.0, indicating language that is similar to spoken (conversational) English.^26^ Terms in the glossary included jargon that the patient may come across during an appointment with a clinician. There were 9 sentences with the passive voice, primarily used to explain the trial findings. Although this number is higher than the recommendation of no more than 1 passive voice in a text, 6 of these were used in identical formats (e.g., “XX in 100 people were offered surgery”). The understanding of key messages was also assessed during user testing, and the text was refined to improve clarity.
**Make sure numbers are easy to understand and do not require calculation**	Numbers were primarily presented as icon arrays with accompanying text giving the absolute percentage. This allowed for a comparison of absolute and relative differences.
**Information is broken down into short sections with informative headings**	The decision aid was broken down into sections that relate to before, during, and after the appointment with the surgeon, with further subheadings within each of these sections (Table 2).
**Health-literate format:** white space, large font size, visual cues for key points; use summaries	White space was prioritized. The recommended font size of 12 was used.^25^ A 1-page infographic was incorporated to succinctly summarize the key information.
**Break down actions into explicit steps**	The values-clarification task was broken down into several steps. It first asks users to reflect on different personal values and then asks users to think about how these values collectively align or do not align with decision options. After that, the user was asked to make a decision, and the text describes the next steps.
**Skills for question asking**	To foster the confidence and skills needed to engage more deeply with decision making, the decision aid has a section that reminds users about their right to asks questions, with general examples of what patients might ask (e.g., asking the doctor to explain something if you don’t understand). There is also a question prompt list designed specifically for people considering surgery for sciatica (e.g., How long will I have to wait for the surgery?). The resource also advises to think ahead about which questions are most important.
**Skills for reflecting on and sharing values**	At each stage of the decision aid, users are reminded to think about and share what matters most to them. The values-clarification tool was designed to support reflection on personal values. Patient stories sought to provide examples of how and why people make different decisions about getting surgery for sciatica.

a Based on topics listed in the PEMAT^24^ and Muscat et al.^9^ core health literacy skills.

The general structure is shown in Table 2. The final version of the decision aid including the PDF version and clinician user guide can be viewed at https://www.sydneyhealthliteracylab.org.au/decision-aid-for-patients-considering-surgery-for-sciatica. It was made available as a word document (for screen readers), as a PDF (for printed versions), and online. The interactive values-clarification component was available only in the online version. QR codes were added to the PDF version to allow people to easily access the online version if needed.

**Table 2:** Patient Decision Aid Structure

Component	Description
Introduction	Describes the target patient group Overview of the different sections (before, during, and after the visit to the surgeon) Link to clinician user guide explaining the purpose of the decision aid, target patient group, details of the scientific evidence, and relevant guidelines
**Before you visit the surgeon**	
What is sciatica?	Describes what sciatica is, feels like, and natural course
What are my options?	Describes the 2 options and their potential benefits and harms, with risk information where possible Key statistics were conveyed using icon arrays (visual design to support people with low numeracy)
Summary of key information (infographic)	Summarized information about sciatica and treatment options presented earlier
Patient stories	Four stories depicting different values and decisions about whether to have surgery or try other options first Prompt to reflect on personal values
**During the visit to the surgeon**	
What happens when I visit the surgeon?	Prompt to ask questions Prompt to discuss what matters most to you Question prompt list for the surgical appointment
**After you visit the surgeon**	
After you visit the surgeon	Prompt to get a second opinion if needed Prompt to use the values-clarification task Reminder that having surgery is your decision Description of next steps once a decision is made
What matters most to you?	Interactive values-clarification task. Users can move a slider to indicate their values. For example, for the “pain” aspect, users can move the slider toward a statement indicating that pain is not manageable (suggesting surgery) or manageable (suggesting try other options first). As each slider is completed, a dynamic bar graph reorders the bars in descending order of importance, so that the user’s strongest responses are shown at the top (a completed bar graph is shown in Figure 1; a video recording is shown in Appendix 2). If these responses are unbalanced, the treatment option that aligns with responses appears in darkened text. Users can edit the wording for each slider and add their own reasons for preferring a treatment option. The general concept drew on experimental work suggesting that similar formats could improve decision making that aligns with personal values.^6^
Your decision	Users indicate their preferred treatment option. A copy of the bar graph then appears with text summarizing whether their preferred option aligns or does not align with responses in the previous section (Figure 1).
**End matter**	
Learn more	Links to government Web sites (from Australia, United Kingdom, United States) References and explanations of how the references supported information in the decision aid
Common terms	Glossary of terms that patients may encounter with their clinicians
About us	Information about developers, funding, and conflicts of interest

### Participants and Recruitment

To be eligible, clinicians needed to be registered with the Australian Health Practitioner Regulation Agency and have experience managing patients with sciatica. Clinicians were recruited through health network groups including professional mailing lists and Facebook groups. Eligible patients were Australian adults who had current or previous experience of low-back pain or sciatica, with their worst episode causing at least a moderate level of interference in their daily activities. Low-back pain and sciatica are related conditions with similar clinical management recommendations.^27^ People with experience of low-back pain without sciatica were eligible to take part. This sought to increase the pool of potential participants without unduly affecting the generalizability of the results. Patient participants were recruited via referral from participating clinicians and via social media ads.

Both sets of participants were purposively sampled to ensure diversity including for age and gender. For clinicians, we sought participants from different professions involved in the patients’ care (GP, physiotherapist, chiropractor, or surgeon). For patients, we sought to capture perspectives of people with differing levels of education and experiences of sciatica or low-back pain. Having less than a university level of education and being born overseas were used as proxies for potential low health literacy, as both characteristics are associated with lower health literacy levels.^28,29^ Participants were recruited between December 2022 and September 2023.

### Interviews

Each participant took part in a single interview over Zoom. Patients were asked to briefly describe their experience of sciatica/low-back pain; clinicians were asked to describe management and workflow for their patients with sciatica (Appendix 3). Participants then shared their screen and looked at the patient decision aid prototype while “thinking aloud.”^30^ General feedback was provided at the end. Audio-visual data and observation notes were recorded. After each round of interviews comprising 5 to 7 participants, the decision aid was revised to incorporate feedback from the previous round.

### Analysis

Framework thematic analysis and a critical realism approach were used to explore participants’ reflections on the decision aid.^31,32^ This process involved 2 analysts applying a brief interpretation to small sections of data (“coding”). Data were primarily coded inductively rather than deductively (i.e., codes were derived from interpreting the data rather than an existing theory or framework). After both analysts coded transcripts from 2 patients and 2 clinicians, they discussed codes and grouped these into categories to develop preliminary themes. The analysts then applied these themes to the remaining transcripts and charted data into a matrix, with each row representing a participant and each column a subtheme. Themes were iteratively discussed with the team and refined.

Two researchers conducted interviews. Author 1 is a female behavioral scientist and public health researcher with extensive experience in qualitative research methods. She does not hold a clinical qualification. Author 3 is a male physiotherapist and research assistant with 4 y of experience treating patients with sciatica and low-back pain. Analysis also incorporated the perspectives of the full research team, including 3 additional musculoskeletal researchers (physiotherapy and chiropractic backgrounds) and 1 public health and shared decision-making researcher.

### Funding Source

Financial support for this study was provided entirely by the Commonwealth Government of Australia’s National Health and Medical Research Council (NHMRC) grant (the Australia and New Zealand Low Back Pain Research Network [ANZBACK] a Centre of Research Excellence; APP1171459).

## Results

A total of 89 clinicians and 70 patients expressed interest in the study, and 20 of each were purposively selected to take part (Table 3; Appendix 4).

**Table 3:** Participant Characteristics of the Clinicians (*n* = 20) and Patients (*n* = 20)

Clinician Characteristics	*n*	%	Patient Characteristics	*n*	%
Profession			Age (y)		
Physiotherapist	6	30	18–39	7	35
Chiropractor	5	25	40–49	5	25
General practitioner	6	30	50–59	5	25
Surgeon	4	20	≥60	3	15
Gender			Gender		
Man	10	50	Man	8	40
Woman	10	50	Woman	11	55
Years’ experience with low-back pain patients			Prefer not to say	1	5
<5	3	15	Current pain experience^a^		
5–9	7	35	Back pain only	6	30
≥10	10	50	Back pain and sciatica	12	60
Country of birth			No current pain	2	10
Australia	11	55	Past pain experience^a^		
Other	9	45	Back pain only	8	40
Setting			Back pain and sciatica	12	60
Private	15	75	Education		
Public	2	10	Less than university	11	55
Urban	15	75	University	9	45
Regional/rural	4	25	Country of birth		
			Australia	11	55
			Other	9	45

a Four participants had experience only of back pain.

Three themes describe 3 broad concepts that participants discussed when interacting with the decision aid and that the authors felt had relevance beyond the decision aid’s specific clinical context. The first theme, “building the case for personal choice,” describes how participants felt that the decision aid incrementally helped them understand the role of personal values and circumstances in the decision to have surgery for sciatica and helped them reflect on their own personal values. The second theme, “cultivating question asking,” explores participant reflections on how the decision aid might help them feel more comfortable and confident interacting with a surgeon. The third theme, “making evidence compelling,” discusses the importance of providing information that participants felt was useful and reliable, in a manner that they could easily understand.

### Theme 1: Building the Case for Personal Choice

Participants discussed that a key strength of the resource was its ability to convey that the decision to have surgery for sciatica was a personal one:

[The patients] recognize . . . at the end of the day it’s their decision . . . it’s not your physio’s, or chiro’s or surgeon’s decision . . . our job is to give you the best information we can for you to come to that decision. (Physiotherapist, 5–9 y of experience, woman)

I think it’s good the way it’s got kind of—ask questions or consider your options because it makes it—it makes a person realize that there are options, there isn’t only one way to go about things. (Patient, 50–59 y, woman, on initially opening the document)

Participants felt this key message was important given that some patients may not recognize that they could be involved in the decision making:

A lot of people think that after talking to the surgeon, they’ve committed [to surgery]. (Chiropractor, 10+ y of experience, man)

This message about personal choice was repeatedly reinforced, for example, by providing relatable patient stories that described varied experiences. After reading the 4 stories, the following patient reflected:

My general thoughts are like different people have different reasons. They decide to get surgery or decide not to, and it really depends on their life and their environment. Like this person [in the story] . . . decided to get surgery because it was like improvement sooner rather than later, and I’m guessing it’s because they’re younger and still have more responsibilities. Yeah, it really depends on the person, what they decide. (Patient, 40–49 y, man)

Some took this concept further and began to think about how similar or different their own experiences were from the people in the stories. One young person explained:

I mean, for me, it’s like, well, isn’t it a bit worrying that I’m only 18 and I’ve got this pain? But it’s good that there are those perspectives there, and you can get a sense of where you fit into with others. (Patient, 18–39 y, woman)

The interactive values-clarification activity also helped reinforce the concept of personal choice and encouraged reflection. Participants discussed how this activity moved them beyond passively reading the text to a deeper engagement with the decision-making process.

I think the thing I liked the most was definitely the interactive feelings one . . . because it’s interactive. Because it’s kind of what really visualizes the person’s factors to decide. And it’s also very, I guess, personal. Like it really connects the user with the Web site, rather than just reading text. (Patient, 50–59 y, man)

Some participants described how putting a number to a personal value helped them reflect on the relative importance of those values:

You’re having to be accountable for how far you’ve put it down the slider, that’s a reflection of how you actually feel. . . . It makes you think about, “Was I being honest? Why did I give this a bit more weighting than that? Is that true? Do I need to have another crack at it with true emotion or whatever?” I think it was definitely helpful in that way. (Patient, 18–39 y, man)

A few participants expressed a preference for recording their values in a binary format (yes/no button). Nevertheless, they were still able to report their personal values by pulling the marker to either end of the slider.

**Figure 1:**
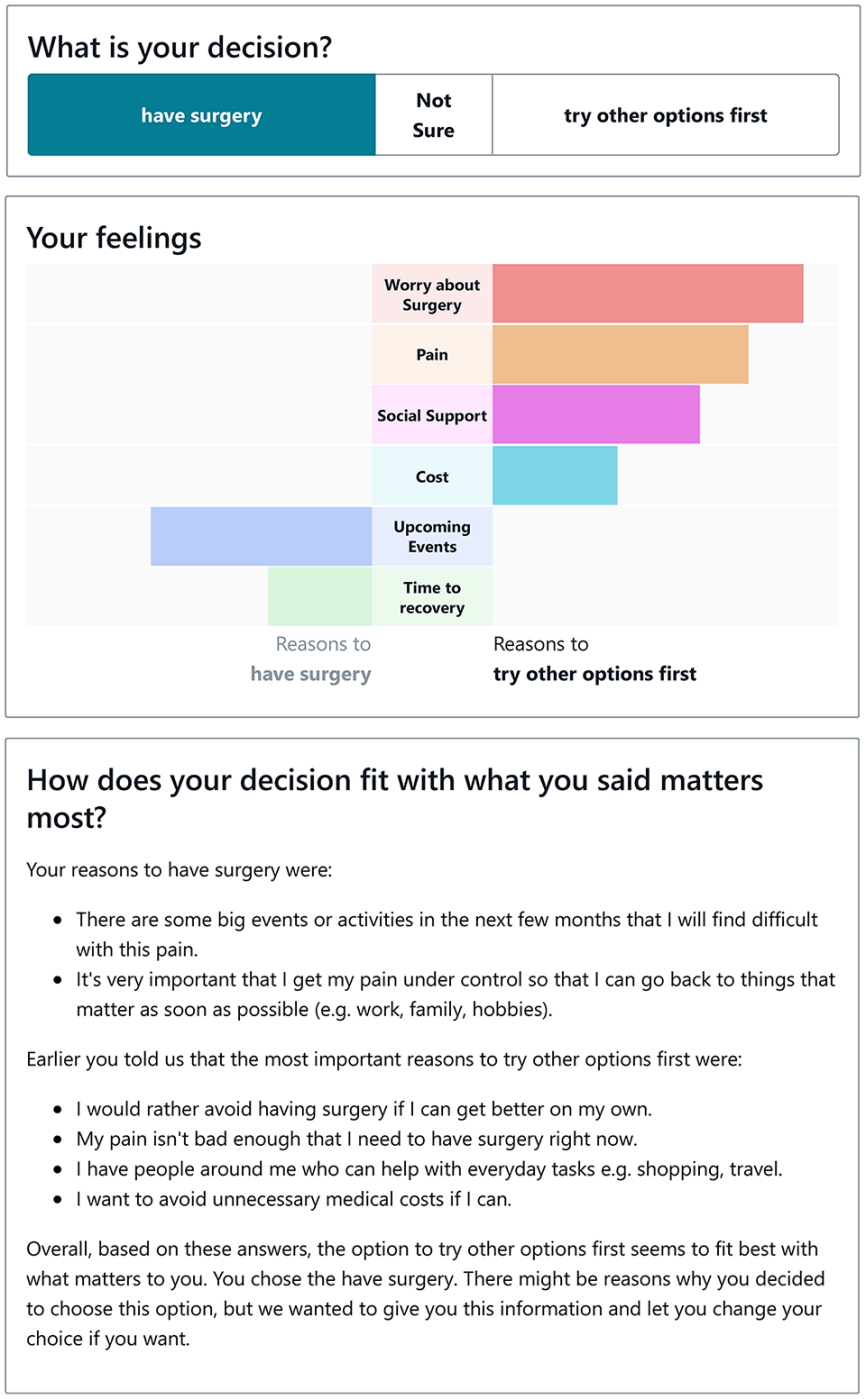
Values-clarification task. Example feedback on the “Your decision” page. In this case, the decision to have surgery does not align with the user’s reported values.

After completing the values-clarification activity, patients were asked what their decision might be. This was followed by a text and visual summary of their stated values (Figure 1). Participants could flick between a values-congruent and values-incongruent decision to observe how the text-based advice would change. Participants appreciated the text that appeared when a user selected a values-incongruent decision and felt that the tone was respectful and appropriate and upheld the overarching message about personal choice:

By using the information that I’ve given personally about my experience, it’s quite reasonable to say that it’s leaning more towards one side . . . I think it’s also good that you say “there might be reasons why you decided to choose this option, but this is what we think.” . . . I think it’s good wording and it’s not like too dramatic at all, and very reasonable.” (Patient, 18–39 y, woman)

Clinician discussions about the interactive values-clarification task were also positive. Many agreed that the tool could help their patients integrate multiple competing values and preferences:

I think it sort of balances all the issues that people are considering, and you know—nobody can handle 6 layers of decision making, and this helps with that. (Surgeon, 10+ y of experience, man)

A potential additional benefit was that the bar graph visualization could help them better understand a patients’ values and needs during an appointment:

It shows you . . . my biggest worry is cost . . . or my biggest worry is pain . . . it’s a good way to visually see their thoughts. (Chiropractor and physiotherapist, 10+ y of experience, man)

### Theme 2: Cultivating Question Asking

Clinicians and patients welcomed text that encouraged question asking. On a practical level, many participants described that the list of questions could help patients prepare, prioritize, and remember to ask important questions during a consultation:

They can even then highlight which questions they would want to ask without having to ask everything and having to come up with it on the spot because I know—I have anxiety, going to a doctor, my brain goes blank. (Patient, 18–39 y, woman)

More generally, advice to ask questions helped participants feel more empowered and highlighted a personal responsibility to reflect on their information needs:

It gives you a lot of power, rather than thinking that they’re the be all and end all, that they know what’s best for you. . . . It’s making you think about what you need to and want to ask before you go. (Patient, 40–49 y, woman)

Participants also highlighted that preparing questions in advance may not be enough. The decision aid also encourages patients to, for example, ask the surgeon to explain terms, repeat or write down what was said, and help them check that they have understood correctly. Clinicians and patients felt these messages were valuable:

There’s a big power imbalance and the patients may be a little bit overwhelmed by the experience. If the surgeon is not using particularly patient-friendly language, then things might start to deteriorate very quickly in terms of the communication. (Chiropractor, 10+ y of experience, man)

Love this square here, right to ask questions. Yep, just because they study for a million years and this is all what they do, doesn’t mean that you can’t ask . . . you’re allowed to be confident and go, “help me.” (Patient, 40–49 y, woman)

Lastly, clinicians were pleased to see advice about getting a second opinion, highlighting that this also helped patients understand that the decision to have surgery was ultimately theirs:

Definitely, absolutely, really good idea. . . . Any ethical surgeon would say, “If you’re not sure then go back and talk to your GP. Maybe get a second opinion.” (Surgeon, 10+ y of experience, man)

Second opinion, great. I love that . . . surgery is invasive, it’s a big thing . . . like they [patients] have the right because it’s their body . . . and so I always make it really clear to people that if they’re not happy with the surgeon . . . they still have the right to say no. (Physiotherapist, less than 5 y of experience, woman)

### Theme 3: Making Evidence Compelling

In addition to fostering a sense of personal choice and capacity to ask questions, most patients discussed how certain decision aid features made the evidence more useful and compelling. For example, patients spoke positively about information they perceived as complete, balanced, and easy to understand:

It’s nice to see everything in one area. I was getting so many different bits of feedback from so many different places that it was kind of hard to just. . . you know . . . put everything in one spot. (Patient, 18–39 y, woman)

I like that it gives you the positives and negatives. . . . I’d be more frightened if they didn’t have the reasons to avoid it. (Patient, 50–59 y, man)

Visual design could also convey a sense of completeness. The following participant appreciated the design that showed recovery rates for the 2 treatment options side by side:

Not just 2 mo after [surgery] . . . the whole timeline, that supporting evidence . . . really good that there’s somewhat consistency and having data from both sides of the spectrum to compare. (Patient, 18–39 y, woman)

Many of the patients described the statistics from research studies as crucial information that would weigh into their decision making. However, some distinguished between information they felt was designed for them and information they felt was primarily targeting clinicians or researchers. For statistics to be compelling and useful, patients felt it was important that they were presented in a manner that was informative and did not overwhelm:

[The decision aid is] actually well-written and easy to read. It’s not overwhelming with data which I find sometimes some of the medical Web sites have got too much reference material in it which is fine if you’re a doctor, but if you’re a layman like myself you don’t need that. (Patient, 50–59 y, woman)

Few patients explicitly spoke about the scientific quality of the evidence. Discussions about scientific quality tended to highlight that logos of well-known universities and the presence of a reference list (without strong intention to read the references) were indicators of quality that helped them trust the information:

You gotta back it up. It’d be useless if you didn’t have anything like this [reference list]. People were saying, “well, you haven’t even shown where you got this information from.” (Patient, 50–59 y, man)

By contrast, clinicians often focused on appraising the scientific validity of the evidence. For example, clinicians felt less confident in studies that were older and that they perceived as having a small sample size, and they felt more confident when evidence was based on systematic reviews of the literature. Many clinicians felt that details about the studies (e.g., sample size, journal publication, relationship to systematic reviews) were best placed in the clinician user guide only and would not be useful for their patients. Some expressed concerns that including such details in the decision aid could potentially confuse or overwhelm patients and unintentionally weaken the key messages. For example, one clinician discussed that there was no need for a reference list (“the patients normally won’t read it,” GP, 5–9 y experience, woman); another expressed that “to understand scientific literature is beyond lay people . . . that academic message is for us” (surgeon, 10+ y of experience, man).

## Discussion

This study explored patient and clinician perceptions of health-literate and values-clarifications features that may be useful in guiding future decision aid development. Patients and clinicians appreciated repeated reinforcement of key messages about personal choice. In particular, both groups felt the values-clarification activity would improve personal reflection and the quality of discussions between patients and clinicians. Patients and clinicians also appreciated strategies that encouraged question asking or getting a second opinion. Lastly, patients described how information felt more compelling when it was perceived as balanced and easy to understand and made reference to reputable information sources.

Patient-centered communication is essential for shared decision making.^33^ In this study, this was enhanced through encouragement to ask questions and the values-clarification task, both of which complemented other health literacy principles incorporated into the decision aid’s design. Collectively, these features supported the goal of fostering the knowledge, confidence, and health literacy skills needed for shared decision making.^9^ For example, findings suggested that the values-clarification task could both help patients reflect on which values mattered most and help clinicians understand and talk to a patient about their values. The first of these findings is supported by experimental research. Compared with other values-clarification methods, tools that explicitly show patients how their values align or do not align with decision options can reduce conflicted feelings about a health decision and increase decision making that aligns with personal values.^6,7^ Our findings showed 2 additional benefits: the values-clarification task may be able to facilitate discussions with clinicians because they can “see” their patient’s values; second, the values-clarification task, in combination with patient stories, may help reinforce to patients that personal values and circumstances really do matter when making health decisions. This study also showed that patients and clinicians value explicit encouragement to ask questions and supported scaffolding to build question-asking skills. This aligns with existing evidence suggesting that this kind of encouragement is particularly important when there is a strong power imbalance between patients and clinicians, such as between a patient and a surgeon.^34–37^

Accurate, evidence-based information is also an essential component of shared decision making.^33^ However, clinicians and patients must also trust the evidence that is presented to them. Systematic reviews have shown that people in the general population trust and prefer health information they perceive as complete, understandable, and unbiased; that comes from reputable organizations; and that include references.^38–41^ In the current study, we used guidelines for risk communication, health literacy, and decision aid design to achieve this,^5,8,19–25^ with many patient participants appreciating content that they felt was complete and easy to understand. To avoid overwhelming patients, we placed some of the more technical information in the clinician user guide. Our study findings align with those of the previous systematic reviews, suggesting that information perceived as easy to understand and balanced is also considered to be of higher quality and more useful.^38–41^ Study findings were also consistent with a review’s suggestion that the presence of references may cultivate trust in health information, even if the reader does not intend to look at them in close detail.^39^

The study has several strengths and limitations. Framework analysis ensured that the 2 primary analysts could easily share and discuss patterns in the data related to each theme. Purposive sampling captured perspectives from a diverse group of clinicians and patients, including a wider range of clinical specialties than other studies of patient decision aids.^17^ However, this was not exhaustive, and study findings may not reflect the perspectives of other clinicians such as rheumatologists and neurologists. Further, we did not directly measure the health literacy of patient participants and relied on related variables (education and speaking another language at home) to purposively sample participants who are likely to have varied health literacy needs.^28,29^ We are not able to determine the health literacy of these participants nor explicitly compare feedback from people with varying health literacy levels. The decision aid is unlikely to be suitable for participants with very low functional health literacy (e.g., low reading ability), given the amount of text present in the decision aid. Further research is needed to evaluate how the decision aid is used in real-world settings, including analyses that explore its appropriateness for people with low health literacy. For example, it is possible that although patients value the decision aid’s features that encourage question asking, these are not sufficient to overcome the power imbalance with a surgeon, particularly in time-limited settings. Similarly, although clinicians anticipated that the values-clarification task would help facilitate discussion about values, we did not observe this directly in this study. Further work is needed to evaluate the extent to which these key features may enhance the design of decision aids in varied clinical contexts.

## Conclusion

Clinicians and patients reported appreciating elements of a health-literate approach to decision aid design and an interactive values-clarification tool. They reported feeling that these features might improve a patient’s capacity to engage in key aspects of shared decision making, including an understanding of personal autonomy in the choice at hand and confidence to ask the clinician questions. Patients also reported that information they perceived as complete and easy to understand and that referenced reputable information sources felt more compelling to them. Further research is needed to evaluate how decision aids with these features are used in practice, across a range of clinical contexts, and whether these features can improve patient and clinician practices related to shared decision making.

## Supplemental Material

sj-docx-1-mdm-10.1177_0272989X251334356 – Supplemental material for Exploring Values Clarification and Health-Literate Design in Patient Decision Aids: A Qualitative Interview Study Supplemental material, sj-docx-1-mdm-10.1177_0272989X251334356 for Exploring Values Clarification and Health-Literate Design in Patient Decision Aids: A Qualitative Interview Study by Julie Ayre, Hazel Jenkins, Richie Kumarage, Kirsten J. McCaffery, Christopher G. Maher and Mark J. Hancock in Medical Decision Making

sj-docx-2-mdm-10.1177_0272989X251334356 – Supplemental material for Exploring Values Clarification and Health-Literate Design in Patient Decision Aids: A Qualitative Interview Study Supplemental material, sj-docx-2-mdm-10.1177_0272989X251334356 for Exploring Values Clarification and Health-Literate Design in Patient Decision Aids: A Qualitative Interview Study by Julie Ayre, Hazel Jenkins, Richie Kumarage, Kirsten J. McCaffery, Christopher G. Maher and Mark J. Hancock in Medical Decision Making

sj-pdf-3-mdm-10.1177_0272989X251334356 – Supplemental material for Exploring Values Clarification and Health-Literate Design in Patient Decision Aids: A Qualitative Interview Study Supplemental material, sj-pdf-3-mdm-10.1177_0272989X251334356 for Exploring Values Clarification and Health-Literate Design in Patient Decision Aids: A Qualitative Interview Study by Julie Ayre, Hazel Jenkins, Richie Kumarage, Kirsten J. McCaffery, Christopher G. Maher and Mark J. Hancock in Medical Decision Making

sj-pdf-4-mdm-10.1177_0272989X251334356 – Supplemental material for Exploring Values Clarification and Health-Literate Design in Patient Decision Aids: A Qualitative Interview Study Supplemental material, sj-pdf-4-mdm-10.1177_0272989X251334356 for Exploring Values Clarification and Health-Literate Design in Patient Decision Aids: A Qualitative Interview Study by Julie Ayre, Hazel Jenkins, Richie Kumarage, Kirsten J. McCaffery, Christopher G. Maher and Mark J. Hancock in Medical Decision Making

sj-pdf-5-mdm-10.1177_0272989X251334356 – Supplemental material for Exploring Values Clarification and Health-Literate Design in Patient Decision Aids: A Qualitative Interview Study Supplemental material, sj-pdf-5-mdm-10.1177_0272989X251334356 for Exploring Values Clarification and Health-Literate Design in Patient Decision Aids: A Qualitative Interview Study by Julie Ayre, Hazel Jenkins, Richie Kumarage, Kirsten J. McCaffery, Christopher G. Maher and Mark J. Hancock in Medical Decision Making
