# Supplementary material for: Exploring Values Clarification and Health-Literate Design in Patient Decision Aids: A Qualitative Interview Study
Source: Med Decis Making. 2025 May 14;45(5):510–21. doi: 10.1177/0272989X251334356 (PMC12166136; doi:10.1177/0272989X251334356)
Supplement: sj-docx-1-mdm-10.1177_0272989X251334356 – Supplemental material for Exploring Values Clarification and Health-Literate Design in Patient Decision Aids: A Qualitative Interview Study [file sj-docx-1-mdm-10.1177_0272989X251334356.docx]

# Appendix 1

**Domains 1 and 2 from the Consolidated Criteria for Reporting Qualitative Studies (COREQ)**

Developed from:

Tong A, Sainsbury P, Craig J. Consolidated criteria for reporting qualitative research (COREQ): a 32-item checklist for interviews and focus groups. *International Journal for Quality in Health Care*. 2007. Volume 19, Number 6: pp. 349 – 357

| **No. Item** | **Guide questions/description** | **Notes** |
| --- | --- | --- |
| **Domain 1: Research team and reﬂexivity** | | |
| ***Personal Characteristics*** |  |  |
| 1. Inter viewer/facilitator | Which author/s conducted the interview or focus group? | Interviews were facilitated by Author 1 and 3 |
| 2. Credentials | What were the researcher’s credentials? E.g. PhD, MD | Author 1: B.LibSt (Hons in Psychology) and PhD (Public Health)  Author 3: Bachelor Physiotherapy |
| 3. Occupation | What was their occupation at the time of the study? | Author 1: Postdoctoral research fellow  Author 3: Research assistant, physiotherapist |
| 4. Gender | Was the researcher male or female? | Author 1: Female  Author 3: Male |
| 5. Experience and training | What experience or training did the researcher have? | Author 1: Training and extensive experience in qualitative research methods  Author 3: Training in qualitative research methods |
| ***Relationship with participants*** | | |
| 6. Relationship established | Was a relationship established prior to study commencement? | No direct contact between participant and interviewer prior the interviews |
| 7. Participant knowledge of the interviewer | What did the participants know about the researcher? e.g. personal goals, reasons for doing the research | Participants were informed that the research team was interested in testing out an online tool to help people with sciatica think about the decision to have surgery or not. Participants knew which universities the researchers were affiliated with. |
| 8. Interviewer characteristics | What characteristics were reported about the inter viewer/facilitator? e.g. Bias, assumptions, reasons and interests in the research topic | None |

| **Domain 2: Study design** |  |  |
| --- | --- | --- |
| ***Theoretical framework*** |  |  |
| 9. Methodological orientation and Theory | What methodological orientation was stated to underpin the study? e.g. grounded theory, discourse analysis, ethnography, phenomenology, content analysis | Methods in this study were based on framework analysis, as outlined in the methods section. |
| ***Participant selection*** |  |  |
| 10. Sampling | How were participants selected? e.g. purposive, convenience, consecutive, snowball | Participants were purposively sampled as stated in the methods. |
| 11. Method of approach | How were participants approached? e.g. face-to-face, telephone, mail, email | Clinicians were recruited through health network groups including professional mailing lists and Facebook groups. Patient participants were recruited via referral from participating clinicians and via social media ads. |
| 12. Sample size | How many participants were in the study? | There were 40 participants in the study. |
| 13. Non-participation | How many people refused to participate or dropped out? Reasons? | No participants dropped out. |
| ***Setting*** |  |  |
| 14. Setting of data collection | Where was the data collected? e.g. home, clinic, workplace | Data were collected online via Zoom with screenshare |
| 15. Presence of non-participants | Was anyone else present besides the participants and researchers? | Only the participant and researcher were present at the time of the interview. |
| 16. Description of sample | What are the important characteristics of the sample? e.g. demographic data, date | See Table 3 |
| ***Data collection*** |  |  |
| 17. Interview guide | Were questions, prompts, guides provided by the authors? Was it pilot tested? | Interviews were semi-structured with a think-aloud protocol. More details are in the methods. |
| 18. Repeat interviews | Were repeat inter views carried out? If yes, how many? | No repeat interviews were carried out. |
| 19. Audio/visual recording | Did the research use audio or visual recording to collect the data? | All interviews included audio-visual recording. |
| 20. Field notes | Were ﬁeld notes made during and/or after the interview or focus group? | Observation notes were made during the interviews, particularly as participants used the online tool. |
| 21. Duration | What was the duration of the inter views or focus group? | Clinician interviews lasted approximately 30 minutes  Patient interviews lasted approximately 45 minutes |
| 22. Data saturation | Was data saturation discussed? | Rather than data saturation, this study used the concept of information power to determine when sufficient data was collected. That is, the data collected was sufficiently rich and deep to answer the research question. |
| 23. Transcripts returned | Were transcripts returned to participants for comment and/or correction? | Transcripts were not returned to participants for comment and/or correction. |
| **Analysis and findings** | | |
| 24. Number of data coders | How many data coders coded the data? | 2 (Author 1 and 3) |
| 25. Description of the coding tree | Did authors provide a description of the coding tree? | No as this is not required for reporting of Framework analysis |
| 26. Derivation of themes | Were themes identified in advance or derived from the data | Derived from the data |
| 27. Software | What software, if applicable, was used to manage the data? | Excel |
| 28. Participant checking | Did participants provide feedback on the data? | No |
| **Reporting** |  |  |
| 29. Quotations presented | Were participant quotations presented to illustrate themes/findings? | Yes |
| 30. Data and findings consistent | Was there consistency between the data presented and the findings | Yes |
| 31. Clarity of major themes | Were major themes clearly presented in the findings? | Yes |
| 32. Clarity of minor themes | Is there a description of diverse cases or discussion of minor themes? | No minor themes. Themes include descriptions of outlier perspectives/experiences. |
