# Supplementary material for: Exploring Values Clarification and Health-Literate Design in Patient Decision Aids: A Qualitative Interview Study
Source: Med Decis Making. 2025 May 14;45(5):510–21. doi: 10.1177/0272989X251334356 (PMC12166136; doi:10.1177/0272989X251334356)
Supplement: sj-docx-2-mdm-10.1177_0272989X251334356 – Supplemental material for Exploring Values Clarification and Health-Literate Design in Patient Decision Aids: A Qualitative Interview Study [file sj-docx-2-mdm-10.1177_0272989X251334356.docx]

# Appendix 6

**Table A1. Clinician characteristics**

| **ID** | **Round of user-testing** | **Profession** | **Gender** | **Country of birth** | **Professional experience (years)** | **Setting** |
| --- | --- | --- | --- | --- | --- | --- |
| CLIN001 | 1 | Chiropractor | Male | Australia | 10+ | Urban, private |
| CLIN002 | 1 | Chiropractor | Female | South Africa | 10+ | Urban |
| CLIN003 | 1 | Chiropractor | Female | Malaysia | 10+ | Urban, private |
| CLIN004 | 1 | Physiotherapist | Female | South Africa | <5 | Urban, private |
| CLIN005 | 1 | Physiotherapist and chiropractor | Male | Australia | 10+ | Urban, private |
| CLIN006 | 2 | Chiropractor | Female | Canada | <5 | Urban, private |
| CLIN007 | 2 | Physiotherapist | Male | Australia | 5‒9 | Urban, private |
| CLIN008 | 2 | Physiotherapist | Male | Australia | 5‒9 | Urban, private |
| CLIN009 | 3 | General practitioner | Female | Australia | 5‒9 | Urban |
| CLIN010 | 3 | Physiotherapist | Male | Australia | 5‒9 | Regional, private |
| CLIN011 | 3 | Physiotherapist | Female | Australia | 5‒9 | Regional/rural, private |
| CLIN012 | 3 | Surgeon | Male | Australia | 10+ | Urban, public |
| CLIN013 | 3 | Surgeon | Male | India | 10+ | Private |
| CLIN014 | 4 | General practitioner | Female | Australia | 10+ | Regional, private |
| CLIN015 | 4 | General practitioner | Female | Australia | 5‒9 | Urban |
| CLIN016 | 4 | General practitioner | Female | Colombia | 5‒9 | Regional |
| CLIN017 | 5 | General practitioner | Female | US | 10+ | Urban, private |
| CLIN018 | 6 | Surgeon | Male | UK | 10+ | Urban, private, public |
| CLIN019 | 6 | Surgeon | Male | UK | 10+ | Urban, private |
| CLIN020 | 7 | General practitioner | Male | Australia | <5 | Urban, private |

**Table A2. Patient characteristics**

| **ID** | **Round of user-testing** | **Age group** | **Gender** | **Pain experience (current)** | **Pain experience (past)** | **Education** | **Country of birth** |
| --- | --- | --- | --- | --- | --- | --- | --- |
| PT001 | 2 | 18-39 | Female | Back pain and sciatica | Back pain only | University education | South Africa |
| PT002 | 2 | 60+ | Female | Back pain and sciatica | Back pain only | University education | Sri Lanka |
| PT003 | 3 | 18-39 | Male | No current pain | Back pain and sciatica | University education | Fiji |
| PT004 | 4 | 40-49 | Male | No current pain | Back pain and sciatica | University education | Australia |
| PT005 | 4 | 40-49 | Female | Back pain and sciatica | No previous pain | University education | Australia |
| PT006 | 5 | 40-49 | Male | Back pain and sciatica | Back pain and sciatica | Less than university education | Australia |
| PT007 | 5 | 60+ | Male | Back pain and sciatica | Back pain only | University education | Australia |
| PT008 | 5 | 50-59 | Female | Back pain only | Back pain only | Less than university education | South Africa |
| PT009 | 5 | 18-39 | Female | Back pain only | Back pain and sciatica | Less than university education | Australia |
| PT010 | 6 | 18-39 | Female | Back pain only | Back pain only | Less than university education | Hong Kong |
| PT011 | 6 | 18-39 | Female | Back pain only | Back pain only | Less than university education | Hong Kong |
| PT012 | 6 | 40-49 | Male | Back pain and sciatica | Back pain and sciatica | Less than university education | Australia |
| PT013 | 6 | 18-39 | Prefer not to say | Back pain and sciatica | Back pain and sciatica | Less than university education | Singapore |
| PT014 | 6 | 18-39 | Female | Back pain only | Back pain only | Less than university education | Vietnam |
| PT015 | 7 | 50-59 | Male | Back pain and sciatica | Back pain and sciatica | University education | India |
| PT016 | 7 | 60+ | Male | Back pain only | Back pain and sciatica | University education | Australia |
| PT017 | 7 | 50-59 | Male | Back pain only | Back pain and sciatica | University education | Australia |
| PT018 | 7 | 50-59 | Female | Back pain and sciatica | Back pain and sciatica | Less than university education | Australia |
| PT019 | 7 | 40-49 | Female | Back pain and sciatica | Back pain only | Less than university education | Australia |
| PT020 | 7 | 50-59 | Female | Back pain and sciatica | Back pain and sciatica | Less than university education | Australia |
