# Supplementary material for: Exploring Values Clarification and Health-Literate Design in Patient Decision Aids: A Qualitative Interview Study
Source: Med Decis Making. 2025 May 14;45(5):510–21. doi: 10.1177/0272989X251334356 (PMC12166136; doi:10.1177/0272989X251334356)
Supplement: sj-pdf-5-mdm-10.1177_0272989X251334356 – Supplemental material for Exploring Values Clarification and Health-Literate Design in Patient Decision Aids: A Qualitative Interview Study [file sj-pdf-5-mdm-10.1177_0272989X251334356.pdf]

Interactive decision aid for patients considering surgery for sciatica:

User-testing study: Interview Schedule

Introduction: *Thank you for agreeing to be interviewed. This study will ask you to try out a new tool to help patients with sciatica who are thinking about surgery. We've designed the tool to help patients prepare for their visit to a surgeon. This means they already have the referral.*

Confidentiality: *Everything you say will be strictly confidential. Your name will be removed from the transcripts.*

Audio and video recording*: I would like to audio-record the interview so I can listen more carefully instead of writing notes. Audio recordings will be transcribed with any identifying names removed. I would also like to record your screen as you use the decision aid resources. You will not be identifiable in the recording, and I will ask you to turn off your camera during that time to maintain your privacy. Is that ok? I will also now rename your Zoom name as a participant ID so that your name is not available in the recording.*

The interview: *The interview will last about [45 minutes for patients, 30 minutes for clinicians]. If there are any questions you don't want to answer just let me know, and you can stop the interview at any time. There are no right or wrong answers. We're really interested in honest feedback! So feel free to give your honest opinion.*

[start recording] Questions – 5 minutes on this section

Part 1. Current experiences with sciatica (5 minutes)

Patients:

1. How long have you had / how long ago did you have sciatica/low back pain?
2. How did you work with your clinician(s) to manage the pain?
3. *If they have had experience of surgery for sciatica/low back pain*: Did you feel that the surgery helped?

Clinicians

1. Can you tell me about your experience delivering care for patients with sciatica? *(prompt for workflow, referrals to surgeons)*

Part 2. User-testing

Introduction
In the next part of the interview, what we'd like you to do is 'think aloud' about what you are doing as you look at the tool, so we can understand the process you went through and what you thought about it.

I won't be able to answer any questions, just do whatever you think is best, and make sure you continue to think aloud the entire time. I will prompt you to keep talking if you are silent for more than 10 seconds. I will also ask you some questions at the end. Does that sound ok? Do you have any questions?

Tool prototype
I'm sending you the link to the tool [Send link to tool through chat]. Please open the tool and share your screen so that I can see it.

Patients:
Imagine you have a referral to see a surgeon about your sciatica. Your doctor suggested you look at this tool to help you prepare. Note that when you open it up, there are three sections, for before, during, and after the visit to the surgeon. As you read through and use the tool, please try to think aloud.

[Ask participant to use the tool. Prompt to keep talking. Other probes in the think aloud tasks may include "What are you thinking now?", "What do you think that means?", "What do you think that's there to do?", "What do you expect to see next?" "Was this what you expected?", "How do you feel about that?"]

1. Were there any parts of the tool that were hard to understand? Hard to use?

2. Which parts were most helpful?

3. Which parts were least helpful?

4. Is there anything you would change to improve the tool?

Clinicians:
As you interact with the tool, please imagine you have a patient with sciatica has been given a referral to see a surgeon. Try to think aloud as you go.

[Ask participant to use the tool. Prompt to keep talking. Other probes in the think aloud tasks may include "What are you thinking now?", "What do you think that means?", "What do you think that's there to do?", "What do you expect to see next?" "Was this what you expected?", "How do you feel about that?"]

1. Were there any parts of the tool that you liked or didn't like?

2. Which parts were most helpful?

3. Which parts were least helpful?

4. How would the tool fit within your usual workflow?

5. Is there anything you would change to improve the tool?

6. What kind of training or resources would you like to help you use the tool? (e.g. format, length)

[Stop recording]

Clinician recruitment: For this project we will also test the tool with patients who have sciatica or low back pain, or who have experienced this in the past. We are looking for patients whose pain had at least a moderate impact on their daily activities, as this is closest to the kinds of patients who might use the tool. We are asking each clinician who takes part to tell 3 patients about the study and show them the flyer. The flyer QR code will take them to the study information sheet and the consent form. Is this ok?

It is very important that you make sure they know there is no obligation to take part, that you won't know whether they decide to take part or not, and that their decision has no impact on the care they will receive. You can also let the participants know that they will receive a $40 gift voucher if they take part in an interview, as stated in the study information sheet.

All participants: ***Thank you for your time! The final part is a 5-minute survey that will ask you to rate the tool. I will email this to you now along with the gift voucher to thank you for your time [and the recruitment flyer (clinicians only).***
